# Living with a child with MSUD: Psychosocial issues of Filipino parents with a child with maple syrup urine disease

**DOI:** 10.1016/j.gimo.2024.101847

**Published:** 2024-04-16

**Authors:** Ma-Am Joy R. Tumulak, Carmencita D. Padilla, Jose Carlo E. Ongchangco, Mercy Y. Laurino, John Benedict B. Lagarde, Ellen S. Regalado, Augusto V. Legaspi, Elizabeth R. Ventura

**Affiliations:** 1 Institute of Human Genetics, National Institutes of Health, University of the Philippines Manila, Manila, Philippines; 2 College of Medicine, University of the Philippines Manila, Manila, Philippines; 3 University of the Philippines Manila, Manila, Philippines; 4 Fred Hutchinson Cancer Center, Seattle, WA; 5 Department of Academic Innovation and Applied Research, Bow Valley College, Calgary, Alberta, Canada; 6 Department of Psychology, University of the Philippines Diliman, Manila, Philippines

**Keywords:** Filipino parents, Maple syrup urine disease, Newborn screening, Philippines, Psychosocial issues

## Abstract

**Purpose:**

Maple syrup urine disease (MSUD) is a common inborn error of metabolism diagnosed in the Philippines. A family may experience stress, anxiety, sorrow, or feelings of helplessness when a child is diagnosed to have a genetic disorder, which can lead to chronic care and disability. This study aims to explore the psychosocial issues experienced by Filipino parents with children having MSUD.

**Methods:**

This is a descriptive and qualitative study. One-to-one interviews using a semi-structured set of questions were done between the months of November 2015 to March 2016. A total of 12 parents were interviewed. Thematic analysis was used.

**Results:**

The diagnosis of MSUD in a child is, indeed, a stressful event for the family. Parents experienced fear, confusion, and hurt, among other emotions. Having a child with MSUD had a negative impact on their families, especially in terms of financial burden, dietary restriction, and marital conflicts leading to separation. However, some parents reported positive effects, such as increased confidence in one’s abilities to care for the affected child and closer relationships among family members.

**Conclusion:**

A diagnosis of MSUD on the child places considerable caregiver burden on the parents. Findings have important implications for genetic counselors.

## Introduction

Maple syrup urine disease (MSUD, OMIM # 248600) is a common inborn error of metabolism diagnosed in the Philippines.^1^ It is an autosomal recessive disorder, characterized by a deficiency of the branched-chain alpha-keto acid dehydrogenase, which results in the accumulation of branched-chain amino acids leucine, isoleucine, and valine. Among Filipinos, the most common variant is a 4.7-kb deletion in the dihydrolipoamide branched-chain transacylase E2 (*DBT*, HGNC:2698) gene.^2^ Treatment is by lifetime dietary restriction of the branched-chain amino acids. Without treatment, progressive neurological deterioration, and eventually death, can occur. Delayed diagnosis and treatment can cause irreversible mental retardation and other developmental delays in the affected child. On the other hand, early diagnosis and prompt management can give babies a good outcome. However, although affected individuals are now expected to have good prognosis, complications such as ketoacidosis, cerebral edema, and death may occur during times of acute stress, such as when there is an intercurrent illness.^3^, ^4^, ^5^

A family may experience stress, anxiety, sorrow, or feelings of helplessness when a child is diagnosed to have a genetic disorder, which can lead to chronic care and disability.^6^, ^7^, ^8^ Some families experience grief because of the lost opportunity of having a normal child.^9^ Chronic illness disrupts the normal family functioning and changes the member’s roles.^10^ According to Reichman et al^11^ (2008), there can be positive and negative effects of living with a disabled child, and this can affect the entire family, including extended family members. This unique shared experience can affect all aspects of family functioning and, in turn, can affect the health and quality of life of the disabled child and the adjustment of the family to the chronic illness.^12^ The family systems model by Kazak (1989) for adaptation and coping in childhood chronic illness supports this view that an illness in a child affects not only the child but the whole family and family system as well.^13^ Therefore, counseling directed to the family must be used by health professionals.

### Financial burden

A number of studies enumerate the various aspects of caregiver burden, and this includes the financial difficulties.^14^, ^15^, ^16^ As with any disease, MSUD creates a big financial burden on the families. First, regular leucine monitoring, which costs Php 400 (∼USD 7), is done at least once a month, depending on whether the child is well or not. The special medical formula milk costs around Php 3500 (∼USD 60) per can. If a child is admitted, hospitalization costs and medicines are also shouldered by the family. Intravenous lipids given to patients cost Php 3500 (∼USD 60) per pack. Regular follow-up with a metabolic specialist is also required. Check-ups with the specialist also incur expenditures such as transportation and food expenses.

### The Filipino family and disability

The family plays a central role in the lives of Filipinos—self-concept and identities are strongly tied to families.^17^ From birth to death, everything that they do, their successes and failures, and important decisions are always done in the context of the family.

However, Filipinos have limited knowledge and exposure to disabilities. Unless they personally know someone who has a disability, they do not know how to behave or react around disabled persons.^18^ When parents realize that their child has an illness or disability, initial reactions are shock and disbelief.^17^ Parents make their child with disability their priority. They seek the assistance of traditional/faith healers, friends, relatives, and health professionals. All family members are obligated to direct their time, effort, and money to help this child in need.

### Effect of chronic illness on the family

Several studies have explored the different effects of having a child with chronic illness in a family. A study by Cederbaum et al^6^ (2001) found that fear, arising from ambiguities in the diagnosis, and sadness are the primary reactions of family members. Feelings of guilt, anxiety, uncertainty, denial, and stress were also reported.^9^ Families struggled to understand why the disease occurred and whether it will improve or get worse or when their child will die.^9^

Some of the more significant stressors in the family are related to cost and access to medications and hospitalizations.^6^^,^^7^^,^^11^^,^^19^, ^20^, ^21^

Aside from the financial burden, negative effects in the family include physical and emotional demands and the logistical complexities of raising a child with a chronic illness,^11^ mental health strains and lack of freedom,^6^ less time for personal pursuits and other family members,^7^ and negative impact on the marriage, such as higher divorce rates and decreased relationship satisfaction.^10^

However, despite the negative effects, positive effects on the family have also been reported, such as learning about personal strengths and becoming compassionate and patient individuals^6^; broadening of horizons, enhancing family cohesion, and increasing connections to community and religious groups^11^; having a renewed faith in God^7^; and increased closeness and support among family members.^10^

### Objective

The study aims to explore the psychosocial issues experienced by Filipino parents with MSUD children, specifically to describe the different psychosocial needs and concerns, and different problems encountered by them in the care of the MSUD child.

## Materials and Methods

Before the conduct of the study, approval of the research protocol was obtained from the University of the Philippines Manila Research Ethics Board.

This study design is descriptive and qualitative. The phenomenological approach, which is the study of consciousness experience as described in a first-person point of view, was used.^22^ Such qualitative studies seek to elucidate the phenomenon under investigation from the point of view of individuals who have experienced it.^23^ Purposive sampling was used. The parents of the patients were interviewed about their experience and psychosocial issues utilizing a semistructured interview as the research tool (Table 1). The questions were translated into Filipino then translated back to English and pilot tested on the first 2 couples who participated in the study.

**Table 1:** Interview questions for parents with a child affected by MSUD

Question No.	Question
1	What is your understanding of MSUD? a. What kind of disease is it? b. Do you know how it is inherited? c. Are you aware about how this condition is treated?
2	How did you learn about the diagnosis of MSUD in your child?
3	What did you feel when you learned about the diagnosis? a. Why did you feel ___ (parent’s answer)?
4	What did you do upon hearing the diagnosis?
5	Please describe your experience in caring for a child with MSUD. a. If it’s a difficult or easy experience, please explain why. b. Is there a difference between caring for a child with MSUD and caring for a child without MSUD?
6	In what ways has a child with MSUD affected you and your family? a. Did it have a negative impact or positive impact? Kindly explain why.

*MSUD*, maple syrup urine disease.

Participants were recruited via 3 methods: (1) the outpatient genetics clinic of the Philippine General Hospital (PGH)—the primary referral center for MSUD cases in the country, (2) private clinics of 7 geneticists (5 in the National Capital Region, 1 in Cebu, and 1 in Davao), and (3) the MSUD Registry, to which the author requested access. For the patients in the outpatient genetics clinic, the primary investigator approached the parents directly to invite them into the study. For the private clinics of the geneticists, the primary investigator wrote them individually to request for the contact information of their patients with MSUD in compliance with the privacy and confidentiality laws of the country. For the MSUD Registry, the primary investigator wrote a letter to the director of the Institute of Human Genetics, National Institutes of Health, University of the Philippines Manila for access to the list of patients.

The study included parents living in Luzon and acting as primary caregivers of a child diagnosed with MSUD via newborn screening, plasma acid analysis, or urine organic analysis.

Due to logistics and budget constraints, families who live in the Visayas and Mindanao areas were not included in this study. If, however, by chance, the family went to the PGH-outpatient department clinic or to their geneticist’s private clinic, they were invited to participate in the study. If they chose to participate, they were included in the study.

The primary investigator, a doctor by training but a research associate by profession and a student of the Masters of Science in Genetic Counseling at the time of the study, conducted the interviews. She was not involved in the medical care of any of the participants.

Interviews, which lasted from 30 minutes to an hour, were conducted in a private room holding only the primary investigator, the parent, and sometimes the patient. Two parents requested that the interviews be done in their home. Field notes were collected as the questions were answered.

All participants who were invited to the study agreed to participate; there were no participants who declined the interview. Written informed consent was facilitated. The procedure of the study, measures for confidentiality, and possible benefits and risks were explained. Data, such as birthday of the patient, year diagnosed, address, name, sex, religion, educational attainment, occupation, patient type (private or charity), contact information, and whether newborn screening was done, were collected. These data were self-reported by the participants. Number codes were assigned to maintain anonymity of the participants. Interviews, which were recorded, lasted from 30 minutes to 1 hour.

Recorded interviews were transcribed and returned to the respondents for confirmation and correction. To analyze the data, thematic analysis, which involves identification of patterns of meanings and deciding how such patterns can be arranged into themes,^24^ was used. Based on preliminary data analysis, thematic saturation was reached after the sixth interview. No new themes were being generated by this time.

Upon familiarization with emerging themes, transcripts were uploaded to Dedoose, a web-based utility designed for qualitative research methods. Each transcript was read, and initial codes were generated by highlighting meaningful patterns and ideas. Interview excerpts or quotes for generated codes were also collected at this stage.^25^ After each transcript was highlighted for codes, potential themes were created by reviewing the excerpts and sorting the codes into groups that could be possible themes. The codes and themes were checked by a second person (ie, a research assistant) to ensure accuracy and consistency of the data.

## Results

Interviews were done from November 2015 to March 2016. A total of 12 participants were interviewed, with 11 mothers (mostly homemakers) and 1 father. The demographic data are summarized in Table 2.

**Table 2:** Demographic data of the participants

Demographic Data	Mother (*n* = 11)	Father (*n* = 1)	Total (*N* = 12)
Mean age (years)	29 (range 20-46)	23	28 (range 20-46)
Child (years)	-	-	4 (range 0.5-22)

Educational Attainment			
College graduate	4 (36%)	-	4 (33%)
College undergraduate	3 (27%)	1 (100%)	4 (33%)
High school graduate	4 (36%)	-	4 (33%)

Occupation			
Homemaker	9 (82%)	1 (100%)	10 (83%)
Bank employee	1 (9%)	-	1 (8%)
Insurance agent	1 (9%)	-	1 (8%)

Religion			
Roman Catholic	10 (91%)	1 (100%)	11 (92%)
Born-Again Christian	1 (9%)	-	1 (8%)

Type of Patient			
Charity	9 (82%)	1 (100%)	10 (83%)
Private	2 (18%)	-	2 (17%)

Newborn Screening Done?			
Yes	-	-	11 (92%)
No	-	-	1 (8%)

Three themes emerged regarding the participants’ experience in raising a child with MSUD: (1) initial reaction of parents upon learning the diagnosis, (2) impact of the MSUD diagnosis on the family, and (3) the difference in raising a child with MSUD. Aside from these 3, no other minor themes emerged. Table 3 summarizes the themes.

**Table 3:** Themes that emerged from the parents of children with MSUD

Initial Reaction of Parents upon Diagnosis	Impact of the MSUD Diagnosis on the Family	Difference in Raising a Child with MSUD
Fear Confusion Hurt Blame Depression Inability to understand Guilt Denial Shock	Positive Closer or stronger family relationship Understanding your partner more Negative Financial concerns Lifestyle restrictions Diet restrictions Conflict in the family Separation with husband Stigma Resigned from work	Special diet or food Special attention given Gets sick easily Slow or delayed development More difficult

*MSUD*, maple syrup urine disease.

### Initial reaction to MSUD diagnosis

The diagnosis of MSUD can be a traumatic event in the family. The parent’s initial reaction upon learning the diagnosis of their child was asked. Common initial reactions were fear, confusion, and hurt.

Seven out of the 12 respondents said that their initial reaction was fear. Participant 10 verbalized her fear by saying (quotations are translated from Filipino), “I was afraid because I read somewhere that MSUD will poison my child’s brain and that she will die.”

Six respondents said they were confused upon diagnosis. Participant 7 verbalized her confusion: *“*It really hurts. It’s very confusing, I didn’t know what to do.”

Five parents confided that they felt hurt. Participant 3 said, “It is quite painful to hear that your child is sick.”

Other responses included: depression, blame, guilt, inability to understand, shock, and denial.

### Impact of MSUD diagnosis in the family

Majority of the participants answered that the disease had a negative impact on their family. A recurring theme among the parents’ responses was increased financial burden: “We were very affected,” participant 8 said. “For instance, every time we go to PGH for our check-up, we incurred a lot of expenses for food and transportation. This money could have been used for food for the family.”

Other reasons portraying that MSUD had a negative impact in the family were the dietary restrictions, lifestyle restrictions (ie, cannot expose the child to the environment because they easily get sick), conflict in the family, stigma, and a mother (participant 12) was forced to resign from her work to take care of the child. Two mothers (participants 1 and 9) reported that the challenges of raising a child with MSUD led to separation. As participant 9 verbalized: *“*We got separated last January. He left us. Maybe because it was financially hard for him (to have a sick child)…”

However, 3 participants answered that the disease had a positive impact. For example, participant 10 said, “It’s normal for siblings to argue. But now, we became closer because they help me in my situation.”

Participant 7 also claimed that because of her child’s illness, she and her husband was able to understand each other better: “We give our baby our full support. We understand each other better now.”

### Difference in raising a child with MSUD

The parent’s experiences in raising a child with MSUD were asked whether it was more or less difficult compared with raising a child without MSUD. All parents agreed that raising a child with MSUD was different and more difficult compared with raising a normal child, in terms of the special diet requirements, special attention given to the child, and their delayed development.

Participant 8 said: “Yes, it is more difficult to take care of our baby with MSUD. Our first child is healthy and has never been hospitalized, by God’s grace, even up to now. My oldest child can eat anything. That’s the biggest difference.”

Participant 11 also verbalized: “We are having difficulty because our baby has to take special foods. Everything has to be measured to make sure leucine levels will not go up.”

## Discussion

The findings have shown that families diagnosed with a child with MSUD have similar experiences and psychosocial issues. Upon initial diagnosis of MSUD, the strongest reactions of the parents were fear, confusion, and hurt, consistent with other studies.^6^^,^^7^^,^^9^

Living with a genetic condition involves the feeling of “losing a normal child” and grief reactions are commonly felt.^26^ These feelings of loss are inevitable in these kinds of diseases but can also give rise to positive psychological reactions, such as increased strength and compassion.^26^ In this study, the participants only mentioned negative psychological reactions upon learning the diagnosis of their child. However, it cannot be discounted that the positive reactions may develop over time, as the family adapts to their situation. For parents, the diagnosis of an illness in their child is a traumatic event; hence, their comprehension of the disease does not always happen immediately after the initial encounter.^27^

When a diagnosis of a genetic condition is made, one of the first reactions of a parent of an affected child is to inquire about the condition and why it occurred. The parent’s question of “why?” reflects their need to understand the cause and a search for meaning.^28^ This search for meaning is an important early task in adaptation to a disease.^29^ Hence, counselors should facilitate the family’s adaptation to the disease by carefully, even repeatedly, educating the family about the disease.

A disease may have a positive and/or negative impact in the family.^6^^,^^7^^,^^10^^,^^11^ The results of this study showed that the impact of MSUD is mostly negative, especially in terms of diet restriction of the child, financial burden of the disease, and marital discord.

In the Philippines, the special milk formula is given for free to the patients. This helps alleviate the financial burden on families. The parents expressed gratefulness for this assistance to them. Despite this, all parents agreed that raising a child with MSUD is more difficult compared with raising a normal child. The difficulty stems from the diet restrictions, their delayed development, and the fact that the child gets sick more easily compared to other children.

These findings highlight the financial and emotional aspects of caring for a child with MSUD. The health care providers, including the genetic counselor, should keep in mind these issues and problems and address them in an effective and prompt manner.

### Implications on genetic counseling

Genetic counseling is defined by the National Society of Genetic Counselors (2006) as “the process of helping people understand and adapt to the medical, psychological, and familial implications of genetic contributions to disease.”^30^ This definition also encompasses the process of family and medical history interpretation, risk assessment, education about the disease, and counseling to promote informed choices.

Genetic counselors, together with the team of health care professionals, can assist the family to understand the disease by having regular follow-up visits with the family, educating the family, assessing their understanding at each visit, and correcting any misconceptions. Through this process of sharing and visiting the family, counselors can help the family find meaning in the illness experience and foster integration of the scientific explanation of illness with other causal explanations.^28^

A study by Rutherford et al^31^ (2014) has shown increased patient adherence to their medical management if a genetic counselor was involved in the initial genetics visit. A crisis-counseling model may be done in the initial phase, right after the diagnosis. Crisis counseling provides a short-term, focused, and specialized approach to support families after a traumatic experience, such as the diagnosis of a child with a genetic condition. It is an important first step to identify the strengths, weaknesses, and resources of the family as they go through the crisis situation.^32^ It supports the families who will go through changes brought about by the diagnosis of illness in their child, and allows them to express themselves about how they feel about the diagnosis.^32^ Through this kind of counseling, genetic counselors can help the family identify ways to gain control of the situation and overcome their fear and confusion. As this study’s findings reveal, educating the family about the condition is important in their acceptance of the situation. Genetic counselors should also assess the parents’ capacity to understand genetic information and adjust the delivery of information in a way that facilitates understanding and coping. This must be a priority in counseling families who just received a diagnosis. Other ways of gaining control are assisting the parents in disclosing the condition to other family members, helping them make treatment or management decisions, and emphasizing the family’s strengths.^28^

After addressing the initial stage of the illness by a crisis-counseling model, long term follow-up should also be done by the counselor. The use of the reciprocal-engagement model can be helpful in this stage.^33^ In this model, the patient and the genetic counselor work together to come up with decisions on their medical management and thus become empowered. Empowered patients are more satisfied and adhere better to their treatment plan.^31^ This model also emphasizes that a genetic counselor should help families feel supported, build a collaborative and trusting relationship, and strive to understand a family’s values and beliefs.

Financial burden is one of the biggest impacts of MSUD on the family. A genetic counselor can assist the family in this regard by referring the family to a social worker, charitable institution, or support group that can assist them in some of their medical needs. The counselor can also refer the family to special organizations that provide free services, such as physical or speech therapy. In this instance, the counselor also serves as a case manager of the patient, who can facilitate referrals to other specialties or foundations as needed.

Another aspect that must be emphasized is the importance of maintaining a good relationship between the parents. The counselor can assist couples in recognizing their strengths in the face of their child’s illness and encourage them to identify coping strategies that have helped them in difficult situations in the past (eg, taking a break from caregiving and spending time together). The counselor can help recognize issues between the couple or among family members and recommend couple’s or family counseling if needed.

### Conclusion

The findings of this study reflect the complex issues and problems of families with a child affected by MSUD. Future research may explore the father’s side (because most of the participants were mothers), the sibling’s experience, identification of coping strategies that families use, and studying the experiences of families with a member having other metabolic diseases.

## Data Availability

Transcripts are available upon request from the corresponding author, but these may be redacted to protect the privacy of the participants. Should these be needed, unpublished short segments of interviews may be made available to other investigators for purposes of verifying or contextualizing conclusions.

## Conflict of Interest

The authors declare no conflicts of interest.
